# Testing Similarity of Parametric Competing Risks Models for Identifying Potentially Similar Pathways in Healthcare

**DOI:** 10.1002/sim.10243

**Published:** 2024-10-12

**Authors:** Kathrin Möllenhoff, Nadine Binder, Holger Dette

**Affiliations:** ^1^ Institute of Medical Statistics and Computational Biology (IMSB) University of Cologne Cologne Germany; ^2^ Institute of General Practice/Family Medicine, Medical Center and Faculty of Medicine University of Freiburg Freiburg Germany; ^3^ Department of Mathematics Ruhr University Bochum Bochum Germany

**Keywords:** bootstrap, multistate models, parametric competing risks models, routine clinical data, similarity, small data

## Abstract

The identification of similar patient pathways is a crucial task in healthcare analytics. A flexible tool to address this issue are parametric competing risks models, where transition intensities may be specified by a variety of parametric distributions, thus in particular being possibly time‐dependent. We assess the similarity between two such models by examining the transitions between different health states. This research introduces a method to measure the maximum differences in transition intensities over time, leading to the development of a test procedure for assessing similarity. We propose a parametric bootstrap approach for this purpose and provide a proof to confirm the validity of this procedure. The performance of our proposed method is evaluated through a simulation study, considering a range of sample sizes, differing amounts of censoring, and various thresholds for similarity. Finally, we demonstrate the practical application of our approach with a case study from urological clinical routine practice, which inspired this research.

## 1 Introduction

Identifying similar healthcare pathways is crucial to increasing the efficiency and quality of healthcare and improving patient outcomes. A healthcare pathway is generally defined as the journey a patient undertakes from their initial contact with a health professional, such as a general practitioner, through referrals to specialists or hospitals, until the completion of treatment for a specific condition. This pathway serves as a timeline that records all healthcare‐related events, including diagnoses, treatments, and any subsequent consultations or hospital readmissions. The recent accessibility of routine medical data, particularly in a university clinical setting, specifically allows to uncover common clinical care pathways, that is, typical sequences of clinical interventions or hospital readmissions. In doing so, it should be recognized that the risks of events occurring in the pathway may vary over time. This paper focusses on an important statistical aspect in this regard: the utilization of flexible parametric competing risks models to test for similar treatment pathways across different patient populations.

Competing risks models, a special case of multistate models [1, 2], offer a sophisticated means to dissect and understand the intricacies of patient healthcare journeys. These models not only track transitions between different health states but also allow for a nuanced analysis of whether different treatment steps still lead to similar subsequent transitions. This research seeks to leverage these models to test for similarities in healthcare pathways, with the overarching goal of enhancing clinical decision‐making. In this regard, we are particularly interested in deciding whether two competing risks models can be assumed to be *similar*, or, in other words, *equivalent*. Once similarity has been established, clinical decision making can profit a lot of this knowledge. Specifically, our work is motivated by the clinical question of pathway similarity between two groups of prostate cancer patients who either received a prior in‐house diagnostic test before surgery or not, and for which we consider their risk of hospital readmission due to several causes. Our primary objective is to assess from routine clinical data whether these risks are similar such that the respective pathways could be combined. From a clinical point of view, the risks for hospital readmission after surgery should not be contingent upon the precise nature of the preceding diagnostic procedure. So, a clinician might assert that, from a clinical perspective, such distinctions should be inconsequential. To rigorously address such scenarios, we aim to develop a sophisticated methodological approach based on competing risks multistate models to statistically validate the similarity of patient pathways.

The theory of competing risks and broader multistate models has a long and rich history, characterized by advancements in mathematical theory and biostatistics. These developments, primarily driven by clinical applications, are extensively summarized in various textbooks and educational articles [2, 3, 4, 5, 6]. Specifically with regard to competing risks analyses, some classical hypothesis testing approaches have been proposed to determine whether there is sufficient evidence to decide in favor of an alternative hypothesis that significant differences exist between groups [7, 8, 9, 10]. However, the opposite, that is, the assessment of the similarity of two groups in a framework of competing risks, has hardly been addressed in the literature to date. For the simplest case, the classical two‐state survival model, several methods are available. The traditional approach of an equivalence test in this scenario is based on an extension of a log‐rank test and assumes a constant hazard ratio between the two groups [11]. However, this assumption, which is rarely assessed and often violated in practice as indicated by crossing survival curves [12, 13], has been generally criticized [14, 15]. As an alternative, Com‐Nougue et al. [16] introduce a nonparametric method, based on the difference of the survival functions and without assuming proportional hazards. In addition, a parametric alternative has recently been proposed by Möllenhoff and Tresch [17], who consider a similar test statistic, but assume parametric distributions for the survival and the censoring times, respectively. However, while their approach does not require an assumption of proportionality, unlike the procedures above, it considers only one particular event and does not take into account competing risks.

Recently, Binder et al. [18] extend the considerations on similarity testing to competing risks models by introducing a parametric approach based on a bootstrap technique introduced earlier [19]. They propose performing individual tests for each transition and conclude equivalence for the whole competing risks model if all individual null hypotheses can be rejected, according to the intersection union principle (IUP) [20]. Their approach, while effective, has some areas for improvement. First, with an increasing number of states the power decreases substantially, as the IUP is rather conservative [21]. Second, their approach builds on the assumption of constant transition intensities, that is, exponentially distributed transition times, which can sometimes be to simplistic (as discussed in, e.g., works by Hill, Lambert, and Crowther [22] and von Cube, Schumacher, and Wolkewitz [23]) Therefore, exploring more flexible methods will typically offer a more fitting model for the underlying data.

The method presented in this paper improves both of these aspects. First, it allows for any parametric model, meaning in particular time‐dependent transition intensities, and these parametric distributions can vary across transitions, resulting in a very flexible modeling framework. Second, we propose another test statistic, which results in one global test instead of combining individual tests for each state and thus results in higher power. The paper is structured as follows. In Section 2, we define the modeling setting, outline the algorithmic procedure for testing the global hypotheses, and provide a corresponding proof of the new test procedure. In Section 3, we demonstrate the validity of the new approach and compare its performance to the previous method [18]. Finally, in Section 4, we explain the application example that inspired this research. Thereby, we particularly highlight the need to consider flexible parametric models whose specific estimators motivate further evaluations of the new method. Finally, we close with a discussion.

## 2 Methods

### 2.1 Competing Risk Models and Parameter Estimation

Following Andersen et al. [3], we consider two independent Markov processes

(1) $${\left(X^{\left(\ell \right)}\left(t\right)\right)}_{t\geq 0}\;\left(\ell =1,2\right)$$

with state spaces {0,1,…,k} to model the event histories as competing risks for samples of two different populations ℓ=1,2. The processes have possible transitions from state 0 to state j∈{1,…,k} with transition probabilities

(2) $${\mathbb{P}}_{0j}^{\left(\ell \right)}\left(0,t\right)=\mathbb{P}\left(X^{\left(\ell \right)}\left(t\right)=j|X^{\left(\ell \right)}\left(0\right)=0\right)$$

Every individual starts in state 0 at time 0, that is, P(X(0)=0)=1. The time‐to‐first‐event is defined as stopping time T=inf{t>0|X(t)≠0} and the type of the first event is X(T)∈1,…,k. The event times can possibly be right‐censored, so that only the censoring time is known, but no transition to another state could be observed. In general, we assume that censoring times C are independent of the event times T. Let

(3) $$\alpha_{0j}^{\left(\ell \right)}\left(t\right)=\lim_{\Delta t\to 0}\frac{{\mathbb{P}}_{0j}^{\left(\ell \right)}\left(t,t+\Delta t\right)}{\Delta t}$$

denote the cause‐specific transition intensity from state 0 to state j for the ℓth model. The transition intensities, also known as cause‐specific hazards, completely determine the stochastic behavior of the process. Specifically, ${\mathbb{P}}_{00}^{\left(\ell \right)}\left(0,t\right)=\exp\left(-\sum_{j=1}^{k}\int_{0}^{t}\alpha_{0j}^{\left(\ell \right)}\left(u\right)\mathrm{du}\right)=\mathbb{P}\left(T^{\left(\ell \right)}\geq t\right)=S^{\left(\ell \right)}\left(t\right)$ denotes the marginal survival probability, that is the probability of not experiencing any of the k events prior to time point t.

We here consider parametric models for the intensities, that is $\alpha_{0j}^{\left(\ell \right)}\left(t\right)=\alpha_{0j}^{\left(\ell \right)}\left(t,\theta_{0j}^{\left(\ell \right)}\right)$, where

(4) $$\theta_{0j}^{\left(\ell \right)}={\left(\theta_{0j1}^{\left(\ell \right)},\ldots ,\theta_{0{\mathrm{jp}}_{j}}^{\left(\ell \right)}\right)}^{⊤}$$

denotes a $p_{j}$‐dimensional parameter vector specifying the underlying distribution. Typical examples of parametric event‐time models are given by the exponential, the Weibull, the Gompertz or the log‐normal distribution, just to mention a few (see e.g., Kalbfleisch and Prentice [24]). Except for the exponential distribution, the intensities vary over time, which makes the estimation procedure more complex compared to the situation of constant intensities.For deriving the likelihood function to obtain estimates ${\hat{\theta }}_{0j}^{\left(\ell \right)}$ of the parameters in (4), we consider possibly right‐censored event times of individuals and assume that two independent samples $X_{1}^{\left(1\right)},\ldots ,X_{n_{1}}^{\left(1\right)}$ and $X_{1}^{\left(2\right)},\ldots ,X_{n_{2}}^{\left(2\right)}$ from Markov processes (1) are observed over the interval 𝒯=[0,τ], each containing the state and transition time (or the censoring time, respectively) of an individual i in group ℓ. Thus, we observe $X_{i}^{\left(\ell \right)}=\left({\tilde{T}}_{i}^{\left(\ell \right)},X^{\left(\ell \right)}\left({\tilde{T}}_{i}^{\left(\ell \right)}\right)\right)$, where ${\tilde{T}}_{i}^{\left(\ell \right)}=\min\left(T_{i}^{\left(\ell \right)},C_{i}^{\left(\ell \right)}\right)$, $i=1,\ldots ,n_{\ell }$. The total number of individuals is given by $n≔n_{1}+n_{2}$.

Following Andersen, Abildstrom, and Rosthøj [1], in case of Type I censoring, that is, a fixed end of the study given by τ, each individual i contributes a factor to the likelihood function given by $S\left(C_{i}\right)$, whereas if there was a transition to state j at time $T_{i}$ the factor would be $S\left(T_{i}\right)\alpha_{0j}\left(T_{i},\theta_{0j}\right)$ (group index ℓ omitted here). Consequently the corresponding likelihood function in the ℓth group, based on $n_{\ell }$ independent observations, is given by the product

(5) $$\begin{array}{l} {ℒ}_{\ell }\left(\theta^{\left(\ell \right)}\right)=\prod_{i=1}^{n_{\ell }}S^{\left(\ell \right)}\left({\tilde{T}}_{i}^{\left(\ell \right)}\right)\prod_{j=1}^{k}\alpha_{0j}^{\left(\ell \right)}{\left({\tilde{T}}_{i}^{\left(\ell \right)},\theta_{0j}^{\left(\ell \right)}\right)}^{I\left\{X^{\left(\ell \right)}\left({\tilde{T}}_{i}^{\left(\ell \right)}\right)=j\right\}} \end{array}$$

where

(6) $$\theta^{\left(\ell \right)}={\left({\left(\theta_{01}^{\left(\ell \right)}\right)}^{⊤},\ldots ,{\left(\theta_{0k}^{\left(\ell \right)}\right)}^{⊤}\right)}^{⊤}$$

is the $p≔\sum_{j=1}^{k}p_{j}$‐dimensional parameter vector specifying the underlying distributions and hence the transition intensities $\alpha_{0j}^{\left(\ell \right)}\left(t\right)$. As ${\tilde{T}}_{i}^{\left(\ell \right)}=T_{i}^{\left(\ell \right)}$, if individual i had a transition to any of the k states, we get, taking the logarithm of (5),

(7) $$\begin{array}{l} \log{ℒ}_{\ell }\left(\theta^{\left(\ell \right)}\right)=\sum_{i=1}^{n_{\ell }}\log\left(S^{\left(\ell \right)}\left({\tilde{T}}_{i}^{\left(\ell \right)}\right)\right)\;\\ \;+\sum_{i=1}^{n_{\ell }}\sum_{j=1}^{k}I\left\{X^{\left(\ell \right)}\left(T_{i}^{\left(\ell \right)}\right)=j\right\}\log\left(\alpha_{0j}^{\left(\ell \right)}\left(T_{i}^{\left(\ell \right)},\theta_{0j}^{\left(\ell \right)}\right)\right) \end{array}$$

By maximizing the functions $\log{ℒ}_{1}$ and $\log{ℒ}_{2}$ in (7) we obtain ML estimates ${\hat{\theta }}^{\left(1\right)}$ and ${\hat{\theta }}^{\left(2\right)}$, respectively.

In case of random right‐censoring, we assume that the censoring times C follow a particular distribution with density g=g(t,ψ) and distribution function G=G(t,ψ), where ψ denotes the parameter specifying the censoring distribution. Technically, assuming random right‐censoring is incorporated in the likelihood as adding an additional state to the model. Precisely, if an individual i is censored at censoring time $C_{i}$, the contribution to the likelihood is given by $\mathbb{P}\left({\tilde{T}}_{i}=C_{i},X\left({\tilde{T}}_{i}\right)=0\right)=\mathbb{P}\left({\tilde{T}}_{i}=C_{i},T_{i}>C_{i}\right)=S\left(C_{i}\right)\cdot g\left(C_{i}\right)$ and thus the likelihood in (5) is extended by an additional factor and, in group ℓ, becomes

(8) $$\begin{array}{l} {ℒ}_{\ell }\left(\theta^{\left(\ell \right)},\psi^{\left(\ell \right)}\right)=\prod_{i=1}^{n_{\ell }}S^{\left(\ell \right)}\left({\tilde{T}}_{i}^{\left(\ell \right)}\right)g^{\left(\ell \right)}{\left({\tilde{T}}_{i}^{\left(\ell \right)},\psi^{\left(\ell \right)}\right)}^{I\left\{X^{\left(\ell \right)}\left({\tilde{T}}_{i}^{\left(\ell \right)}\right)=0\right\}}\\ \;\prod_{j=1}^{k}\alpha_{0j}^{\left(\ell \right)}{\left({\tilde{T}}_{i}^{\left(\ell \right)},\theta_{0j}^{\left(\ell \right)}\right)}^{I\left\{X^{\left(\ell \right)}\left({\tilde{T}}_{i}^{\left(\ell \right)}\right)=j\right\}} \end{array}$$

and, accordingly, the log‐likelihood in (7) becomes

(9) $$\begin{array}{l} \log{ℒ}_{\ell }\left(\theta^{\left(\ell \right)},\psi^{\left(\ell \right)}\right)=\sum_{i=1}^{n_{\ell }}\log\left(S^{\left(\ell \right)}\left({\tilde{T}}_{i}^{\left(\ell \right)}\right)\right)\\ +\;\sum_{i=1}^{n_{\ell }}I\left\{X^{\left(\ell \right)}\left({\tilde{T}}_{i}^{\left(\ell \right)}\right)=0\right\}\log g^{\left(\ell \right)}\left({\tilde{T}}_{i}^{\left(\ell \right)},\psi^{\left(\ell \right)}\right)\\ +\;\sum_{i=1}^{n_{\ell }}\sum_{j=1}^{k}I\left\{X^{\left(\ell \right)}\left(T_{i}^{\left(\ell \right)}\right)=j\right\}\log\left(\alpha_{0j}^{\left(\ell \right)}\left(T_{i}^{\left(\ell \right)},\theta_{0j}^{\left(\ell \right)}\right)\right) \end{array}$$

### 2.2 Similarity of Competing Risk Models

An intuitive way to define similar competing risk models is by measuring the maximum distance between transition intensities and decide for similarity if this distance is small. Note that, due to an easier readability, we omit the dependency of the intensities $\alpha_{0j}^{\left(\ell \right)}$ on the parameters $\theta_{0j}^{\left(\ell \right)}$, j=1,…k, throughout the following discussion. Therefore the hypotheses are given by

(10) $$\begin{array}{c} H_{0}:\;\text{there}\;\text{exists}\;\text{an}\;\text{index}\;j\in \left\{1,\ldots ,k\right\}\;\\ \text{such}\;\text{that}\;∥\alpha_{0j}^{\left(1\right)}-\alpha_{0j}^{\left(2\right)}{∥}_{\infty }\geq \Delta \end{array}$$

versus

(11) $$H_{1}:\;\text{for}\;\mathrm{all}\;j\in \left\{1,\ldots ,k\right\}\;∥\alpha_{0j}^{\left(1\right)}-\alpha_{0j}^{\left(2\right)}{∥}_{\infty }<\Delta$$

where Δ is a prespecified threshold and $∥f-g{∥}_{\infty }=\sup_{t\in 𝒯}|f\left(t\right)-g\left(t\right)|$ denotes the maximal deviation between the functions f and g.

Note that the formulation of the hypotheses differs from the “classical” hypotheses $H_{0}:\max_{j=1}^{k}∥\alpha_{0j}^{\left(1\right)}-\alpha_{0j}^{\left(2\right)}{∥}_{\infty }=0$ versus H1:maxj=1k∥α0j(1)−α0j(2)∥∞=0 and has two advantages. First, it is very unlikely that **all** transition intensity functions $\alpha_{0j}^{\left(1\right)}$ and $\alpha_{0j}^{\left(2\right)}$ do exactly coincide. As they correspond to different groups the difference may be very small but probably never exactly equal to 0. This point of view is in line with Tukey, who argued in his paper [25] (in the context of multiple comparisons of means) that … “All we know about the world teaches us that the effects of A and B are always different—in some decimal place—for any A and B. Thus asking “Are the effects different?” is foolish” …. Taking this point of view it might be more reasonable, to ask if the transition intensity functions do not deviate substantially. Second, defining the null hypothesis and alternative as in (10) and (11), respectively, and not in the opposite way, allows to decide for similarity, that is $\max_{j=1}^{k}∥\alpha_{0j}^{\left(1\right)}-\alpha_{0j}^{\left(2\right)}{∥}_{\infty }<\Delta$, at a controlled Type I error.

This test problem can be addressed by two different types of test procedures. If one is interested in comparing each pair of transition intensities $\alpha_{0j}^{\left(1\right)}\left(t\right)$ and $\alpha_{0j}^{\left(2\right)}\left(t\right)$, j=1,…,k, over the entire interval [0,𝒯] individually, we propose to do a separate test for each of these k comparisons and to combine them via IUP [20] as described in Binder et al. [18] This method has the advantage that one can make inference about particular differences between transitions and the threshold in (11) can be replaced by individually chosen thresholds $\Delta_{j}$, j=1,…,k, for each single comparison. However, if the threshold Δ is globally chosen, as stated in (10) and (11), applying the same principle means that the similarity of the *j*th transition intensities is assessed by testing the individual hypothesis

(12) $$H_{0}^{j}:∥\alpha_{0j}^{\left(1\right)}-\alpha_{0j}^{\left(2\right)}{∥}_{\infty }\geq \Delta$$

versus

(13) $$H_{1}^{j}:∥\alpha_{0j}^{\left(1\right)}-\alpha_{0j}^{\left(2\right)}{∥}_{\infty }<\Delta$$

However, combining these individual tests to obtain a global test decision results in a noticeable loss of power, which is a well known consequence of tests based on the IUP [21]. Therefore, if one is interested in claiming similarity of the whole competing risks models rather than comparing particular transition intensities, another test procedure should be considered. This procedure is based on re‐formulating $H_{1}$ in (11) to

(14) $$H_{1}:\;\max_{j=1}^{k}∥\alpha_{0j}^{\left(1\right)}-\alpha_{0j}^{\left(2\right)}{∥}_{\infty }<\Delta$$

which gives rise to another test statistic. Based on this, the following algorithm describes a much more powerful procedure for testing the hypotheses (10) against (14). It is based on a constrained parametric bootstrap generating data under the null hypothesis. However, in contrast to testing a classical null hypothesis of the form $H_{0}:\max_{j=1}^{k}∥\alpha_{0j}^{\left(1\right)}-\alpha_{0j}^{\left(2\right)}{∥}_{\infty }=0$, which defines a single point in the corresponding parameter space, the situation is more complicated, as the hypothesis in (12) defines a manifold in the parameter space. Therefore, there are several possibilities to generate data under the null hypothesis. In Algorithm 1, we generate the data such that the bootstrap data satisfies (asymptotically) the condition $\max_{j=1}^{k}∥\alpha_{0j}^{\left(1\right)}-\alpha_{0j}^{\left(2\right)}{∥}_{\infty }=\Delta ,$ to increase the power.

ALGORITHM 1

1. For both samples, calculate the MLE ${\hat{\theta }}^{\left(\ell \right)}$ and ${\hat{\psi }}^{\left(\ell \right)}$, ℓ=1,2, by maximizing the log‐likelihood given in (9), in order to obtain the transition intensities ${\hat{\alpha }}^{\left(1\right)}$ and ${\hat{\alpha }}^{\left(2\right)}$ with ${\hat{\alpha }}^{\left(\ell \right)}=\left({\hat{\alpha }}_{01}^{\left(\ell \right)},\ldots ,{\hat{\alpha }}_{0k}^{\left(\ell \right)}\right)$ and the parameters ${\hat{\psi }}^{\left(\ell \right)}$, ℓ=1,2, of the underlying censoring distributions. Note that, in case of no random censoring, it suffices to maximize the log‐likelihood in (7). From the estimates, calculate the corresponding test statistic

$$\hat{d}≔\max_{j=1}^{k}∥{\hat{\alpha }}_{0j}^{\left(1\right)}-{\hat{\alpha }}_{0j}^{\left(2\right)}{∥}_{\infty }$$

2 In a second estimation step, we define constrained estimates ${\bar{\theta }}^{\left(1\right)}$ and ${\bar{\theta }}^{\left(2\right)}$ of $\theta^{\left(1\right)}$ and $\theta^{\left(2\right)}$, maximizing the sum $\log{ℒ}_{1}\left(\theta^{\left(1\right)}\right)+\log{ℒ}_{2}\left(\theta^{\left(2\right)}\right)$ of the log‐likelihood functions defined in (7) under the additional constraint
(15) $$\max_{j=1}^{k}∥\alpha_{0j}^{\left(1\right)}-\alpha_{0j}^{\left(2\right)}{∥}_{\infty }=\Delta$$
  Further define
(16) $${\hat{\hat{\theta }}}^{\left(\ell \right)}=\left\{\begin{array}{ccc} {\hat{\theta }}^{\left(\ell \right)} & \;\text{if}\; & \hat{d}\geq \Delta \\ {\bar{\theta }}^{\left(\ell \right)} & \;\text{if}\; & \hat{d}<\Delta \end{array}\right.,\;\ell =1,2$$
  where ${\hat{\hat{\theta }}}^{\left(\ell \right)}={\left({\hat{\hat{\theta }}}_{01}^{\left(\ell \right)},\ldots ,{\hat{\hat{\theta }}}_{0k}^{\left(\ell \right)}\right)}^{⊤}$. From this, we obtain constrained estimates of the transition intensities ${\hat{\hat{\alpha }}}_{0j}^{\left(\ell \right)}\left(t\right)=\alpha_{0j}^{\left(\ell \right)}\left(t,{\hat{\hat{\theta }}}_{0j}^{\left(\ell \right)}\right)$, j=1,…,k, ℓ=1,2. Finally, note that this constraint optimization does not affect the estimation of the censoring distribution.

3 By using the constrained estimates ${\hat{\hat{\alpha }}}^{\left(\ell \right)}={\hat{\hat{\alpha }}}^{\left(\ell \right)}\left(t\right)=\left({\hat{\hat{\alpha }}}_{01}^{\left(\ell \right)}\left(t\right),\ldots ,{\hat{\hat{\alpha }}}_{0k}^{\left(\ell \right)}\left(t\right)\right)$, simulate bootstrap event times $T_{1}^{*\left(1\right)},\ldots ,T_{n_{1}}^{*\left(1\right)}$ and $T_{1}^{*\left(2\right)},\ldots ,T_{n_{2}}^{*\left(2\right)}$. Specifically we use the simulation approach as described in Beyersmann et al. [26], where at first for all individuals survival times are simulated with all‐cause hazard $\sum_{j=1}^{k}{\hat{\hat{\alpha }}}_{0j}^{\left(\ell \right)}\left(t\right)$ as a function of time and then a multinomial experiment is run for each survival time T which decides on state j with probability ${\hat{\hat{\alpha }}}_{0j}^{\left(\ell \right)}\left(T\right)/\sum_{j=1}^{k}{\hat{\hat{\alpha }}}_{0j}^{\left(\ell \right)}\left(T\right)$. In order to represent the censoring adequately, we now use the parameters ${\hat{\psi }}^{\left(\ell \right)}$, ℓ=1,2 from step (i) to additionally generate bootstrap censoring times $C_{1}^{*\left(1\right)},\ldots ,C_{n_{1}}^{*\left(1\right)}$ and $C_{1}^{*\left(2\right)},\ldots ,C_{n_{2}}^{*\left(2\right)}$, according to a distribution with distribution function $G^{\left(1\right)}\left(t,\psi^{\left(1\right)}\right)$ and $G^{\left(2\right)}\left(t,\psi^{\left(2\right)}\right)$, respectively. Finally, the bootstrap samples are obtained by taking the minimum of these times in each case, that is ${\tilde{T}}_{i}^{*\left(\ell \right)}=\min\left(T_{i}^{*\left(\ell \right)},C_{i}^{*\left(\ell \right)}\right)$. Note that, in case of no random but administrative censoring with a fixed end of the study τ, we take ${\tilde{T}}_{i}^{*\left(\ell \right)}=\min\left(T_{j}^{*\left(\ell \right)},\tau \right)$, $i=1,\ldots ,n_{\ell },\;\ell =1,2$.
  For the datasets $X_{1}^{*\left(1\right)},\ldots ,X_{n_{1}}^{*\left(1\right)}$ and $X_{1}^{*\left(2\right)},\ldots ,X_{n_{2}}^{*\left(2\right)}$, consisting of the potentially censored event time and the simulated state of an individual, calculate the MLE ${\hat{\alpha }}^{*\left(1\right)}$ and ${\hat{\alpha }}^{*\left(2\right)}$ by maximizing (7) and the test statistic as in Step (i), that is

(17) $${\hat{d}}^{*}≔\max_{j=1}^{k}∥{\hat{\alpha }}_{0j}^{*\left(1\right)}-{\hat{\alpha }}_{0j}^{*\left(2\right)}{∥}_{\infty }$$

4 Repeat Step (3) B times to generate B replicates of the test statistic ${\hat{d}}^{*\left(1\right)},\ldots ,{\hat{d}}^{*\left(B\right)}$, yielding an estimate of the α‐quantile of the distribution of the statistic $d^{*}$, which is denoted by $q_{\alpha }^{*}$. Finally reject the null hypothesis in (10) if
(18) $$\hat{d}\leq q_{\alpha }^{*}$$
  Alternatively, a test decision can be made based on the p value
$${\hat{F}}_{B}\left(\hat{d}\right)=\frac{1}{B}\sum_{i=1}^{B}I\left\{{\hat{d}}^{*\left(i\right)}\leq \hat{d}\right\}$$
  where ${\hat{F}}_{B}$ denotes the empirical distribution function of the bootstrap sample. Finally, we reject the null hypothesis (10) if ${\hat{F}}_{B}\left(\hat{d}\right)<\alpha$ for a prespecified significance level α.

Depending on the research question one could also want to consider not the entire time range starting at 0 but at a particular $t^{*}>0$, that is, replacing 𝒯=[0,τ] by $𝒯=\left[t^{*},\tau \right]$ in all steps of the test procedure of Algorithm 1. Further, the end of the observational period τ could also be replaced by an earlier time point if the interest is more on earlier phases of the trial. These are very small modifications which do not change any properties of the test. The following result shows that Algorithm 1 defines a valid statistical test for the hypotheses (10) and (14). The proof is deferred to the Appendix.

Theorem 2.1 *Assume that* $\lim_{n_{1},n_{2}\to \infty }n_{1}n_{2}=c>0$ *and that Assumption A‐D in Borgan* [27] *are satisfied*. *Further let*

$$\left\|f{\|}_{\infty ,\infty }≔\max_{j\in \left\{1,\ldots ,k\right\}}\|f_{j}\left(t\right){\|}_{\infty }=\max_{j\in \left\{1,\ldots ,k\right\}\times 𝒯}|f_{j}\left(t\right)\right|$$

*denote the* $\ell^{\infty }$‐*norm on the set of functions* $\left(j,t\right)\to f_{j}\left(t\right)$ *defined on* {1,…,k}×𝒯. *Then the test defined by* (18) *is consistent and has asymptotic level* α *for the hypotheses* (10) *and* (14). *More precisely*,

1. *if the null hypothesis in* (10) *is satisfied*, *then we have for any* α∈(0,0.5)

(19) $$\underset{n\to \infty }{\text{lim sup}}\mathbb{P}\left(\hat{d}\leq q_{\alpha }^{*}\right)\leq \alpha$$

2 *if the null hypothesis in* (10) *is satisfied and the set*

(20) $$ℰ=\left\{\left(j,t\right)\in \left\{1,\ldots ,k\right\}\times 𝒯:|{\hat{\alpha }}_{0j}^{\left(1\right)}\left(t\right)-{\hat{\alpha }}_{0j}^{\left(2\right)}\left(t\right)|=∥{\hat{\alpha }}^{\left(1\right)}-{\hat{\alpha }}^{\left(2\right)}{∥}_{\infty ,\infty }\right\}$$

*consists of one point*, *then we have for any* α∈(0,0.5)

(21) $$\lim_{n\to \infty }\mathbb{P}\left(\hat{d}\leq q_{\alpha }^{*}\right)=\left\{\begin{array}{ccc} 0 & \;\text{if}\; & \max_{j=1}^{k}∥{\hat{\alpha }}_{0j}^{\left(1\right)}-{\hat{\alpha }}_{0j}^{\left(2\right)}{∥}_{\infty }>\Delta \\ \alpha & \;\text{if}\; & \max_{j=1}^{k}∥{\hat{\alpha }}_{0j}^{\left(1\right)}-{\hat{\alpha }}_{0j}^{\left(2\right)}{∥}_{\infty }=\Delta \end{array}\right.$$

3 *if the alternative in* (14) *is satisfied*, *then we have for any* α∈(0,0.5)

(22) $$\lim_{n\to \infty }\mathbb{P}\left(\hat{d}\leq q_{\alpha }^{*}\right)=1$$

Remark 1 An essential ingredient in our approach is the threshold Δ, which defines similarity. Its choice has to be carefully discussed in each application. We can also determine a threshold from the data which can serve as measure of evidence for similarity with a controlled Type I error α.

To be precise, note that the bootstrap statistic in (17) depends on Δ (because the data is generated under the constraint (15)). Therefore we denote in this remark the statistic and corresponding α‐quantile in (18) by ${\hat{d}}_{\Delta }^{*}$ and ${\hat{q}}_{\alpha ,\Delta }^{*}$, respectively. Note also that the hypotheses in (10) and (11) are nested, in the sense that rejection of the null for a particular threshold $\Delta_{1}>0$ implies also rejection for all $\Delta_{2}\geq \Delta_{1}$. It is now easy to see that this monotonicity transfers to the bootstrap statistic in (17), that is ${\hat{d}}_{\Delta_{1}}^{*}\leq {\hat{d}}_{\Delta_{2}}^{*}$. Consequently, we obtain for the corresponding quantiles in (18) the inequality ${\hat{q}}_{\alpha ,\Delta_{1}}^{*}\leq {\hat{q}}_{\alpha ,\Delta_{2}}^{*}$, and rejecting the null hypothesis in (10) by the test in Algorithm 1 for $\Delta =\Delta_{0}$ also yields rejection of the null for all $\Delta >\Delta_{0}$.

Therefore, by the sequential rejection principle, we may simultaneously test the hypotheses in (10) for different Δ≥0 starting at Δ=0 and increasing Δ to find the minimum value ${\hat{\Delta }}_{\alpha }$ for which $H_{0}$ is rejected for the first time. This value could be interpreted as a measure of evidence for similarity with a controlled Type I error α.

## 3 Simulation Study

The goals of the simulations are to validate the Type I error and the power of the hypothesis test proposed in Algorithm 1, and to compare its performance to the previously proposed individual method of Binder et al. [18] First, we present the simulation design, including four different scenarios considered, each determined by the underlying data generating distributions of transition intensities. Second, we present the results, including simulated Type I errors and power, for all four scenarios, assuming different sample sizes and levels of censoring.

### 3.1 Design

We assume two different settings for the distributions of the transition intensities, resulting in four different scenarios in total. All scenarios are driven by the application example given in Section 4 and visualized in Figure 5. In Scenario 1 and Scenario 2, we assume the event times to follow an exponential distribution, that is, all transition intensities are assumed to be constant. This setting is the same as already considered for the simulations in Binder et al. [18] We denote the approach mentioned therein by “Individual Method” throughout the rest of this paper, as it is based on combining three individual tests, one for each state. Consequently, in this setting all results from the two methods are directly comparable. The parameters of the constant transition intensities are given in Table 4 in Section 4, these are used for Scenario 1, yielding

$$d=\max_{j=1}^{3}∥\alpha_{0j}^{\left(1\right)}-\alpha_{0j}^{\left(2\right)}{∥}_{\infty }=\max\left\{0.0002,0.0006,0.0005\right\}=0.0006$$

for Scenario 1. For Scenario 2, we choose identical models, that is $\alpha_{01}^{\left(1\right)}=\alpha_{01}^{\left(2\right)}=0.001,\;\alpha_{02}^{\left(1\right)}=\alpha_{02}^{\left(2\right)}=0.0011$ and $\alpha_{03}^{\left(1\right)}=\alpha_{03}^{\left(2\right)}=0.0004$, respectively, resulting in a difference of 0 for all transition intensities and thus providing the possibility to simulate the maximum power of the procedure.

For the second setting, that is, Scenario 3 and Scenario 4, respectively, we assume a Gompertz distribution for the first two states and a Weibull distribution for the third state, that is, the intensities of the first two states are given by

(23) $$\alpha_{0j}^{\left(\ell \right)}\left(t,\theta_{0j}^{\left(\ell \right)}\right)=\theta_{0j1}^{\left(\ell \right)}\cdot \exp\left(\theta_{0j2}^{\left(\ell \right)}\cdot t\right),\;j=1,2,\;\ell =1,2$$

where $\theta_{0j1}^{\left(\ell \right)}$ denotes the scale and $\theta_{0j2}^{\left(\ell \right)}$ the shape parameter, respectively, and the transition intensity for the third state is given by

(24) $$\alpha_{03}^{\left(\ell \right)}\left(t,\theta_{03}^{\left(\ell \right)}\right)=\frac{\theta_{032}^{\left(\ell \right)}}{\theta_{031}^{\left(\ell \right)}}\cdot {\left(\frac{t}{\theta_{031}^{\left(\ell \right)}}\right)}^{\theta_{032}^{\left(\ell \right)}-1},\;\ell =1,2$$

where $\theta_{031}^{\left(\ell \right)}$ denotes the scale and $\theta_{032}^{\left(\ell \right)}$ the shape parameter, respectively. By assuming these two distributions, this scenario yields a very accurate approximation to the actual data, see Figure 5 in Section 4. More precisely, modeling the transition intensities by the Gompertz and the Weibull distribution, instead of assuming constant intensities, provides a much better initial model fit, resulting in a simulation setup with very realistic conditions with regard to the real data example.

We choose the parameters given by the corresponding transition intensities of the application example (see Table 4 in Section 4), resulting in

$$d=\max_{j=1}^{3}∥\alpha_{0j}^{\left(1\right)}-\alpha_{0j}^{\left(2\right)}{∥}_{\infty }=\max\left\{0.0003,0.0028,0.0004\right\}=0.0028$$

for Scenario 3. Similar to Scenario 2, we obtain Scenario 4 in this setting by considering two identical models, such that $\theta^{\left(2\right)}=\theta^{\left(1\right)}$ and consequently we have d=0 in this case. Of note, Scenario 2 and Scenario 4 can only be used to simulate the power of the test.

In order to simulate the Type I error and the power of the procedure described in Algorithm 1, we consider different similarity thresholds Δ. When simulating Type I errors, we assume Δ=d in both scenarios considered, which reflects the situation at the margin of the null hypothesis. Therefore, we simulate the maximum Type I error. The other values of Δ are chosen so that the differences in simulated power are as clear as possible. Table 1 gives an overview of the simulation scenarios.

**TABLE 1:** Chosen distributions of the simulation scenarios, the resulting maximum distance between transition intensities d and the similarity thresholds Δ under consideration.

	Distribution				
	State 1	State 2	State 3	*d*	Thresholds Δ
Scen. 1	Exp.	Exp.	Exp.	0.0006	**0.0006**, 0.001, 0.0015
Scen. 2	Exp.	Exp.	Exp.	0	0.001, 0.0015
Scen. 3	Gompertz	Gompertz	Weibull	0.0028	**0.002**, **0.0028**, 0.004, 0.005, 0.007, 0.01
Scen. 4	Gompertz	Gompertz	Weibull	0	0.004, 0.005, 0.007, 0.01

*Note*: Numbers in bold correspond to simulations of Type I errors. As d=0 in Scenario 2 and Scenario 4, respectively, we only simulate the power there (Exp. = Exponential).

Based on the application example, where $n_{1}=213$ and $n_{2}=482$ patients are observed in the first and second group, we consider a range of different sample sizes, that is, $n=\left(n_{1},n_{2}\right)=\left(200,200\right),\left(250,300\right),\left(300,300\right),\left(250,450\right),\left(300,500\right)$, and (500,500). Also driven by the application example, we assume administrative censoring with a given follow‐up period of 90 days. Consequently, we consider two competing risk models, each with j=3 states over the time range 𝒯=[0,90]. If there is no transition to one of the three states, an individual is administratively censored at these 90 days.

To additionally investigate the effect of different types of censoring we consider a second setting replacing the administrative censoring by random right‐censoring, where censoring times are generated according to an exponential distribution. Here, the observed time for an individual is given by the minimum of the simulated censoring time and the event time, respectively. By varying the rate parameter of the exponential distribution, we are able to investigate the effect of different amounts of censoring. Precisely, we consider different rate parameters between 0.0002 and 0.01, resulting in approximately 16% up to 85% of the individuals being censored (details for the particular scenarios are given when discussing the results in Section 3.2). For the sake of brevity, when investigating the effect of random censoring, we restrict ourselves to Scenarios 1 and 3 respectively, and three different sample sizes, that is $n=\left(n_{1},n_{2}\right)=\left(200,200\right),\left(300,300\right)$, and (500,500).

The data in all simulations is generated according to the algorithm described in Beyersmann et al. [26] All simulations have been run using R Version 4.3.0. The total number of simulation runs is N=1000 for each configuration and due to computational reasons the test is performed using B=250 bootstrap repetitions. The computation time using an Intel Core i7 CPU with 32 GB RAM for one particular dataset with B=250 bootstrap repetitions is approximately 10 s for Scenarios 1 and 2 and varies between 3 min and 11 min for Scenarios 3 and 4, depending on the sample size under consideration.

### 3.2 Results

#### 3.2.1 Scenario 1

When simulating Type I errors, we assume Δ=d in both scenarios under consideration, reflecting the situation on the margin of the null hypothesis. Thus, in Scenario 1, we set Δ=0.0006.

**FIGURE 1:**
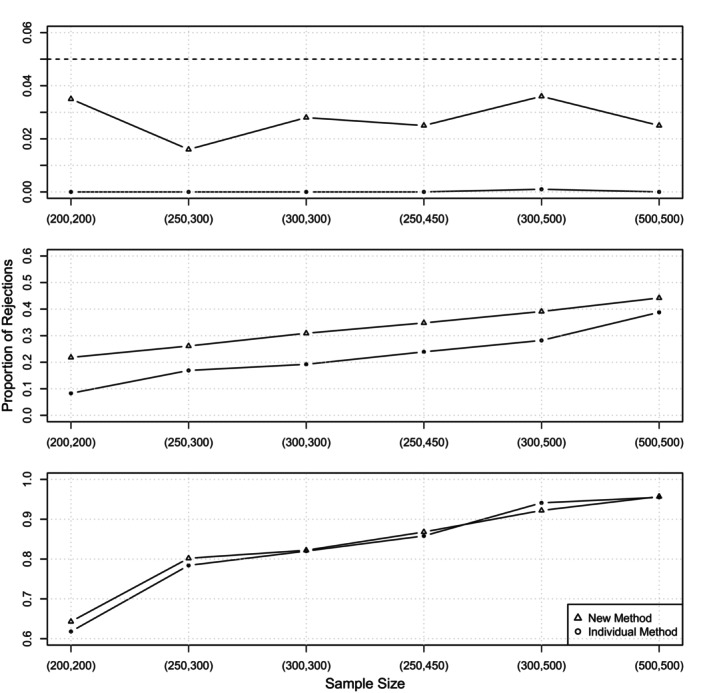
Scenario 1: Proportion of rejections in dependence of the sample size for the new method and the individual method [18]. The three rows display different choices of Δ, that is Δ=0.0006 corresponding to the null hypothesis in the top row, Δ=0.001 in the middle and Δ=0.0015 in the bottom row, where the latter two correspond to the situation under alternative. The dashed line in the first row indicates the nominal level chosen as α=0.05.

First, we consider administrative censoring as described above, that is, a fixed end point of the study at τ=90 days. The first row of Figure 1 displays the Type I error rates of the procedure proposed in Algorithm 1 in dependence of the sample size, directly compared to the ones derived by the “Individual method” presented in Binder et al. [18] (see also Section 2.2). We observe that Type I errors are much closer to the desired level of α=0.05, whereas they are practically 0 for the individual method, where the latter is a direct consequence of the construction based on the IUP, see Section 2.2. The still rather conservative behavior of the test can be explained theoretically: according to Theorem 2.1, we expect Type I errors to be smaller than α, as transition intensities are constant and consequently their differences are constant functions as well, meaning that the set of points maximizing these functions each consists of the entire time range 𝒯.

For the power simulations, we chose two different thresholds in order to also show the relationship between power and the choice of the threshold. As in Binder et al. [18], we chose Δ=0.001 and Δ=0.0015, respectively, but in general all choices of Δ larger than d=0.0006 would reflect a scenario under the alternative (14). The second and third row of Figure 1 visualize the power for both procedures, for Δ=0.001 and Δ=0.0015, respectively. For the latter the difference between the two methods is rather small and only visible for small sample sizes. However, for Δ=0.001 we clearly observe that the power of the new method is higher than the power of the individual method, for all sample sizes under consideration, but in particular for smaller sample sizes.

Regarding the effect of random right censoring, Table 2 displays the results for the simulated Type I error and the power considering different amounts of censoring. Precisely, we consider censoring rates between 0.001 and 0.1, resulting in approximately 22%−80% of the individuals being censored. The first column corresponds to the null hypothesis (10), whereas the last two columns present the power of the procedures for the two different thresholds Δ=0.001 and Δ=0.0015, respectively. The numbers in brackets correspond to the results from the individual procedure [18] for an easier comparability. It turns out that, in contrast to administrative censoring, the new method suffers from some Type I error inflation for low sample sizes if censoring rates become large. For example, for $n_{1}=n_{2}=200$ and a censoring rate of 77%, we observe a Type I error of 0.220, but this scenario means that on average there are only 46 patients per group where a transition to one of the three states is observed, which explains the overly liberal behavior of the test. The opposite holds for the individual procedure which is extremely conservative, as the simulated level is practically zero in all configurations. This Type I error inflation disappears for increasing sample sizes. For instance, considering $n_{1}=n_{2}=500$ all simulated Type I errors are below 0.06, except for a censoring rate of 0.01, corresponding to approximately 80% of the individuals being censored. Hence we conclude that Type I errors still converge to the desired level of α=0.05 with increasing sample sizes.

**TABLE 2:** Scenario 1: Simulated level (Column 4) and power (Columns 5–6) of the new method, that is, the test described in Algorithm 1, considering different sample sizes, censoring rates and thresholds Δ.

$\left(n_{1},n_{2}\right)$	Censoring rate	Censored (%)	Δ=0.0006	Δ=0.001	Δ=0.0015
(200200)	0.001	25	0.037 (0.000)	0.534 (0.476)	0.964 (0.852)
	0.002	40	0.042 (0.000)	0.410 (0.363)	0.916 (0.752)
	0.003	50	0.053 (0.000)	0.373 (0.313)	0.807 (0.656)
	0.005	63	0.107 (0.000)	0.326 (0.226)	0.772 (0.480)
	0.01	77	0.220 (0.000)	0.290 (0.076)	0.535 (0.209)
(300300)	0.001	25	0.045 (0.001)	0.694 (0.618)	0.990 (0.966)
	0.002	40	0.060 (0.000)	0.587 (0.553)	0.965 (0.932)
	0.003	50	0.056 (0.000)	0.504 (0.470)	0.902 (0.853)
	0.005	63	0.083 (0.001)	0.359 (0.356)	0.772 (0.758)
	0.01	77	0.161 (0.000)	0.307 (0.183)	0.562 (0.452)
(500500)	0.001	25	0.050 (0.001)	0.847 (0.831)	1.000 (1.000)
	0.002	40	0.057 (0.000)	0.791 (0.751)	0.987 (0.985)
	0.003	50	0.052 (0.000)	0.685 (0.682)	0.967 (0.976)
	0.005	63	0.060 (0.000)	0.545 (0.583)	0.887 (0.922)
	0.01	77	0.119 (0.000)	0.371 (0.333)	0.664 (0.772)

*Note*: The numbers in brackets correspond to the results from the individual procedure. The nominal level is chosen as α=0.05. The third column displays the mean proportions of censored individuals.

Regarding the power we observe a substantial improvement with the new method for almost all configurations, particularly in case of small sample sizes and large censoring rates, for example, achieving now a simulated power of 0.290 instead of 0.076 for $n_{1}=n_{2}=200$ and a censoring rate of 0.01. If sample sizes are large, the results of both procedures are qualitatively the same which is in line with the asymptotic theory stated in Binder et al. [18] and in Theorem 2.1 of this paper.

#### 3.2.2 Scenario 2

**FIGURE 2:**
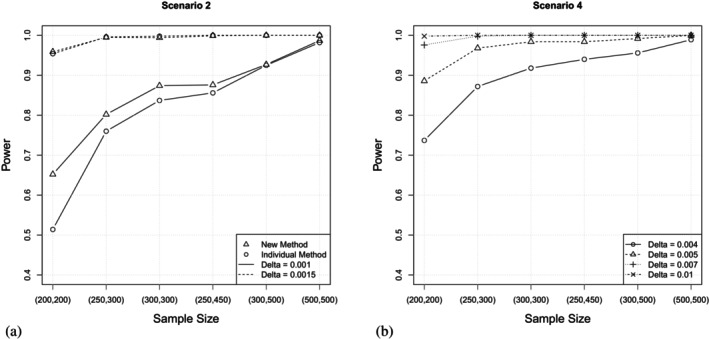
(a) Scenario 2, constant intensities, d=0: Power of the new method and the individual method [18] in dependence of the sample size for different similarity thresholds. (b) Scenario 4, Gompertz and Weibull distributed intensities, d=0: Power of the new method in dependence of the sample size for different similarity thresholds. As Scenario 2 and Scenario 4 assume different underlying distributions, different similarity thresholds are considered for a meaningful analysis.

We still assume constant intensities for all transitions, but choose two identical models, that is $\theta^{\left(2\right)}=\theta^{\left(1\right)}$, resulting in d=0. Consequently, we now simulate the maximum power of the test. Figure 2a displays a direct comparison of the method proposed in Algorithm 1 and the individual method. We observe that for the smaller similarity threshold of Δ=0.001 the power of the new method is higher for all sample sizes under consideration. Of note, this effect is much more visible for smaller sample sizes. For instance, considering $n_{1}=n_{2}=200$ the power is given by 0.652 for the new method and 0.415 for the individual method, respectively, whereas almost identical values (0.982 and 0.987, resp.) are observed for the largest sample size of $n_{1}=n_{2}=500$. Considering Δ=0.0015, the same conclusion can be drawn for larger similarity thresholds, as all values of the simulated power are qualitatively the same across the two methods.

#### 3.2.3 Scenario 3

For simulating the Type I error in Scenario 3, we consider Δ=0.002 and Δ=0.0028, the latter again reflecting the situation of being on the margin of the null hypothesis. Note that for this choice of parameters for each of the three difference curves $\alpha_{0j}^{\left(1\right)}\left(t\right)-\alpha_{0j}^{\left(2\right)}\left(t\right)$, j=1,2,3, the maximum over the time range 𝒯 is attained at one single point. Consequently, the set ℰ defined in Theorem 2.1 consists of this one point, meaning that this simulation scenario reflects the situation in (19).

Again, these theoretic findings are supported by the simulation results, which are displayed in Table 3. Precisely, we observe that Type I error rates converge to the desired level of α=0.05 with increasing sample sizes. For instance, considering the scenario which is the closest to the application example, that is, $\left(n_{1},n_{2}\right)=\left(250,400\right)$ the simulated Type I error is given by 0.059. However, we observe a slight Type I error inflation for the smaller samples under consideration, that is up to 300 patients per group. For example, the highest observed Type I error is given by 0.110, attained for sample sizes of $n_{1}=n_{2}=200$. Of note, for this configuration the number of expected transitions is only 36 for group 1 and 46 for group 2, respectively, due to the high amount of censoring (see also Section 4). The power increases with increasing sample sizes. We note that the threshold should not be too small, as the power is not very satisfying in this case. For instance, we observe a power of 0.2 for a medium sample size of $n_{1}=n_{2}=300$ and a very small threshold of Δ=0.004, whereas it almost doubles for Δ=0.005 and finally approximates 1 for Δ=0.01.

**TABLE 3:** Scenario 3: Simulated level (Column 2) and power (Columns 3–6) of the new method, that is, the test described in Algorithm 1, considering different sample sizes and thresholds Δ. The nominal level is chosen as α=0.05.

$\left(n_{1},n_{2}\right)$	Δ=0.0028	Δ=0.004	Δ=0.005	Δ=0.007	Δ=0.01
(200200)	0.110	0.198	0.316	0.602	0.920
(250300)	0.094	0.194	0.366	0.742	0.978
(300300)	0.080	0.200	0.367	0.756	0.982
(250450)	0.068	0.208	0.426	0.860	0.996
(300500)	0.060	0.196	0.463	0.900	0.996
(500500)	0.055	0.233	0.528	0.920	1.000

**FIGURE 3:**
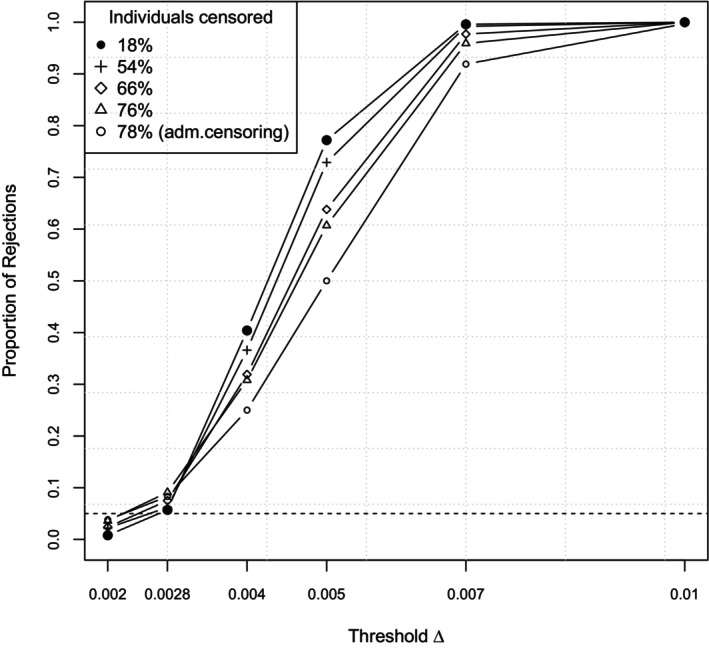
Scenario 3: Proportion of rejections for different amounts of censoring at a fixed sample size of $n_{1}=n_{2}=500$ in dependence of the threshold. The first two thresholds correspond to the null hypothesis (where the second one displays the margin situation), the last four to the alternative. The dashed line indicates the nominal level α=0.05.

Finally, Figure 3 displays the results for the simulated Type I error and the power considering different amounts of random right censoring for a fixed sample size of $n_{1}=n_{2}=500$. Censoring rates are chosen as 0.0002, 0.001, 0.002, and 0.005, resulting in mean proportions of censored individuals ranging from approximately 16% up to 80%. We observe that even for high censoring rates the power is reasonably high and, moreover, higher than in case of administrative censoring at the end of the study. However, this comes at the cost of a slightly inflated Type I error, which attains its maximum of 0.091 for the highest censoring rate of 0.005. When considering administrative censoring, which results in very similar proportions of censored individuals, the corresponding Type I error is given by 0.055, demonstrating that for this type of censoring the problem of Type I error inflation does not occur.

#### 3.2.4 Scenario 4

We now consider two identical models as in Scenario 2, but we assume a Gompertz distribution for the first two states and a Weibull distribution for the third one, respectively. All other configurations remain as described in Scenario 3. Consequently, we thereby simulate the maximum power, as d=0. Figure 2b displays the power of the test in dependence of the sample size for different similarity thresholds Δ. We note that the power is reasonably high and above 0.8 for all configurations except for the combination of the smallest threshold and the smallest sample size.

## 4 Application Example: Healthcare Pathways of Prostate Cancer Patients Involving Surgery

In our application example, we examine coding data from routine inpatient care of prostate cancer patients at the Department of Urology at the Medical Center—University of Freiburg, which was systematically processed as part of the German Medical Informatics Initiative. For each inpatient case, the main and secondary diagnoses are coded in the form of ICD10 codes (10th revision of the International Statistical Classification of Diseases and Related Health Problems); in addition, all applied and billing‐relevant diagnostic and therapeutic procedures are coded together with a time stamp in the form of OPS codes (operation and procedure codes).

Specifically, we consider cases that have undergone open surgery with resection of the prostate including the vesicular glands, also known as open radical prostatectomy (ORP). We retrospectively identified all patients with prostate cancer who underwent ORP at the Department of Urology, University of Freiburg, between January 1, 2015 and February 1, 2021. This resulted in a total of *n* = 695 patients. The current diagnostic standard before such a surgical procedure is a magnetic resonance imaging‐based examination with targeted fusion biopsy (FB). In our data, *n* = 213 (31%) patients received an FB diagnosis prior to ORP, while a larger proportion of patients, *n* = 482 (69%), did not receive an FB diagnosis in the Department of Urology prior to ORP.

**FIGURE 4:**
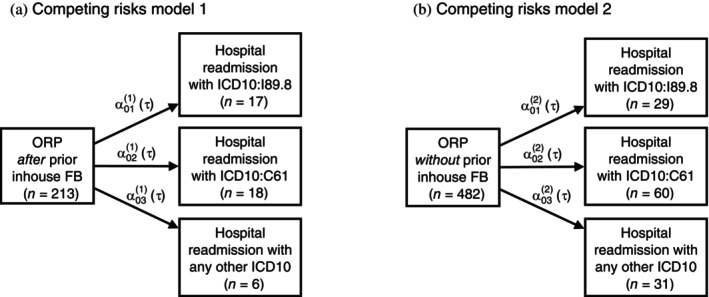
Competing risks multistate models illustrating healthcare pathways for two populations: (A) patients *receiving* inhouse fusion biopsy prior to open radical prostatectomy and (B) patients *not receiving* inhouse fusion biopsy prior to open radical prostatectomy. The arrows indicate the transitions between the states that are investigated. The $\alpha_{0j}^{\left(\ell \right)}\left(t\right)$, j=1,2,3,ℓ=1,2 mark the transition intensities as functions of time (see Equation 3).

In the healthcare pathway after ORP, in some cases there are hospital readmissions due to competing causes, which can be attributed to the surgery in the period of typically 90 days after surgery. The question now is whether these pathways are similar irrespective of the type of prior diagnosis. Therefore, we distinguish two populations, ℓ=1,2, based on the FB diagnosis obtained prior to surgery and aim to investigate the similarity of subsequent pathways using the two independent competing risk models, as shown in Figure 4, where the $\alpha_{0j}^{\left(\ell \right)}\left(t\right),j=1,2,3,\ell =1,2$, describe the transition intensities to the different possible states in the model (see (3)). In the data, the following hospital readmissions occurred over time within 90 days after surgery: Lymphocele (ICD10:I89.9; Model 1: *n* = 17, 8%; Model 2: *n* = 29, 6%), malignant neoplasm of the prostate (ICD10:C61, Model 1: *n* = 18, 8%; Model 2: *n* = 60, 12%), or “any other diagnosis” (Model 1: *n* = 6, 3%; Model 2: *n* = 31, 6%). We administratively censor follow‐up at 90 days after ORP.

**FIGURE 5:**
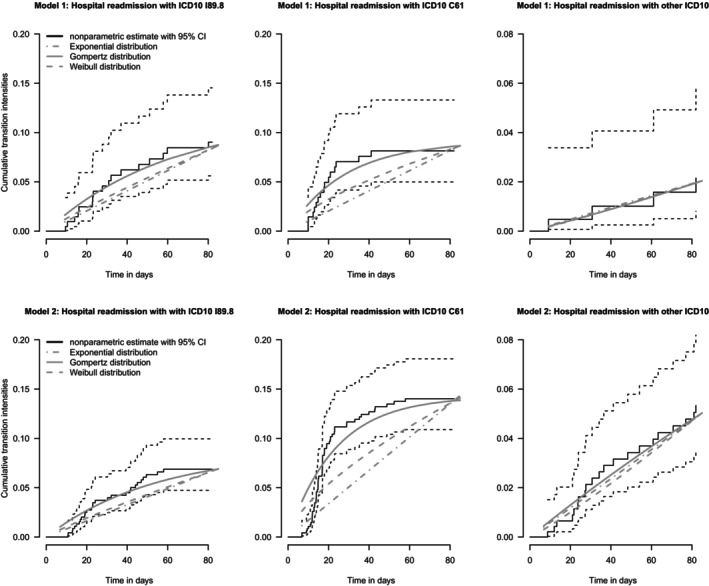
Estimates of the cumulative transition intensities from competing risks Model 1 (upper row), and competing risks Model 2 (lower row) in the application example. Illustrated for each panel are the nonparametric Nelson‐Aalen estimates (black lines) along with 95% confidence intervals (CI), as well as parametric model fits from Gompertz distribution (solid gray lines), Weibull distribution (dashed gray lines), and exponential distribution (dashed‐dotted gray lines).

To understand the dynamics and magnitude of the different risks and to identify a suitable parametric distribution, we estimate the cumulative transition intensities in both models nonparametrically using the Nelson–Aalen estimator [28]. In addition, we fit an exponential, Weibull, and Gompertz model to the data. The estimates are shown in Figure 5. For the first and second competing risks states in both models, the estimates indicate a clear nonconstant accumulated risk, and specifically the Gompertz distribution captures the time dynamics in all cumulative intensities best (as compared with the nonparametric estimates). For the third state, a Weibull fit seems to be equally suitable as a fit from the Gompertz model, even the assumption of constant intensities seems to be met. As overall only few events were observed per state, the magnitude of the transitions intensities is low, and correspondingly the uncertainty of estimates relatively high. This is also reflected in the estimates of the parameters of the transitions intensities (see Table 4).

**TABLE 4:** Estimates of the parameters $\theta_{0j}^{\left(\ell \right)}$ (4) of potential event time distributions for the three transition intensities from competing risks Model 1 and competing risks Model 2 in the application example.

	Model 1			Model 2		
	${\hat{\theta }}_{01}^{\left(1\right)}$	${\hat{\theta }}_{02}^{\left(1\right)}$	${\hat{\theta }}_{03}^{\left(1\right)}$	${\hat{\theta }}_{01}^{\left(2\right)}$	${\hat{\theta }}_{02}^{\left(2\right)}$	${\hat{\theta }}_{03}^{\left(2\right)}$
Exponential	**0.001**	**0.0011**	**0.004**	**0.0008**	**0.0017**	**0.0009**
Gompertz	**0.002, −0.016**	**0.003, −0.036**	0.0002, 0.003	**0.002, −0.018**	**0.006, −0.043**	0.0007, −0.003
Weibull	−0.112, 1304.5	−0.38, 3098.3	**0.097, 2894.8**	−0.12, 1729.8	−0.404, 1595.9	**0.108, 1242.1**

*Note*: For Gompertz and Weibull, the first value corresponds to the scale and the second value to the shape parameter (following 23 and 24). Numbers in bold are used in the simulation study.

**FIGURE 6:**
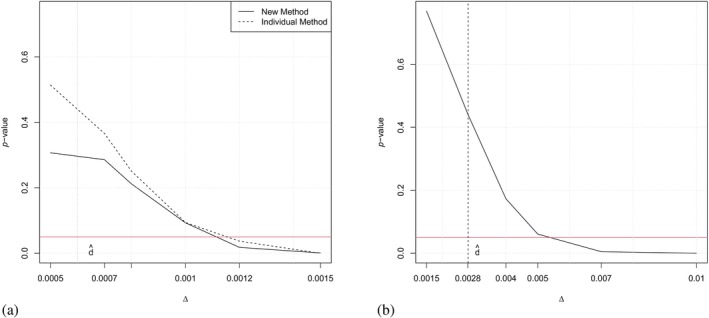
(a) *p* Values of the test described in Algorithm 1 (new method, solid line) compared with the individual method [18] (dashed line) for the application example assuming constant intensities, in dependence of the threshold Δ. (b) *p* Values of the test described in Algorithm 1 assuming a Gompertz/Weibull model in dependence of the threshold Δ. The horizontal line indicates a *p* value of 0.05, the vertical line indicates the test statistic $\hat{d}$.

For investigating the similarity of the two competing risk models using Algorithm 1, we assume two different settings of event time distributions and various similarity thresholds Δ, ranging from 0.0005 to 0.0015. Subsequently, when assuming constant intensities, we will compare the results of this analysis with the results obtained by the individual method [18]. As described in Remark 1, by determining the minimum threshold Δ in a data‐adaptive manner, this Δ serves as a measure of evidence for similarity with a controlled Type I error α. Figure 6 displays the results of the tests in dependence of the similarity threshold Δ, and we can read from the Figure which Δ it is at which we can first reject the null hypothesis. The *p* values for the individual method are obtained by the maximum of the *p* values of the three individual tests, as this is the necessary condition to conclude similarity of the competing risk models [18]. Figure 6a directly yields a comparison of the two methods. As expected, the *p* values of the test proposed in Algorithm 1 are overall similar, but slightly smaller than the ones from the individual method, due to the generally lower power of the latter. Consequently, according to the new method, the null hypothesis can be rejected for a minimal threshold of Δ=0.0011. This means that for at least this threshold similarity of both patient populations regarding all their transition intensities in the model can be claimed. The *p* values in Figure 6b correspond to the more realistic setting of fitting Weibull/Gompertz distributions. We observe that the threshold has to be at least Δ=0.005 such that the null hypothesis can be rejected and similarity of both groups can be claimed. Of note, as the difference of the curves lies on another scale as when assuming constant intensities, these results cannot be compared with the *p* values displayed in Figure 6a.

## 5 Discussion

In this work, we have addressed the question of whether two competing risk models can be considered similar, specifically in situations with fairly small numbers of transitions. Building on the foundation laid by Binder et al. [18], we have extended the approach in two innovative ways. First, we have successfully overcome the previous restriction to constant intensities. Although we have concentrated in the illustrations of Sections 3 and 4 on the exponential, Weibull and Gompertz model, our refined method introduces a framework that can incorporate arbitrary parametric regression models for the transition intensities as considered, for example, in Liu, Pawitan, and Clements [29]. This advance not only allows for a more nuanced modeling of transition intensities, but also leads to a more robust and effective testing procedure. Second, we introduced a novel test statistic: the maximum of all maximum distances between transition intensities. This replaces the earlier method of aggregating individual state tests using the IUP. Through comprehensive simulation studies, we demonstrated the superior power of this new procedure.

While our approach introduces a unified similarity threshold Δ, replacing the need for multiple individual thresholds $\Delta_{j}$, j=1,…,k, this does come with a trade‐off. The loss of detailed information in individual state comparisons is a consideration, but this is balanced by the increased overall power of the test. For those seeking detailed comparisons, individual tests should still be considered. However, for a broader assessment of the similarity between two competing risk models, our new approach is clearly superior. Choosing a global threshold Δ is a necessity of the construction of the test statistic, which now bundles the maximum distances of all transition intensities into one single value d via taking their maximum instead of performing individual tests for each state. An area for future exploration is the challenge of interpreting differences between transition intensities and establishing a meaningful similarity threshold. A potential solution could be to develop a test statistic based on ratios, allowing for more universally applicable thresholds, such as a permissible deviation of 10%. This would simplify the process, accommodating ratios within the range of 0.9−1.1, regardless of the absolute intensity values. For example, such an approach is common in bioequivalence trials, where the threshold is set to log1.25, which results from allowing a deviation of ± 20% and a log‐transformation of the exposure parameters [30].

Since the proposed approach relies on the correct specification of the underlying models, we investigated the robustness by further simulations under different levels of misspecification, again based on the underlying application example. We conclude that, depending on the degree of misspecification of the models, for small to moderate sample sizes both mild Type I error inflation and conservative behavior with loss of power can be observed. However, we find that for moderate levels of misspecification, the simulated values are very close to those obtained from correctly specified models. The detailed results can be found in the Supporting Information. We further note that the simulation study focuses on situations with relatively small numbers of cases and very few transitions, and it could be argued that the proposed test procedure is less useful when much larger amounts of data are available. However, the availability of large amounts of data is of limited use in longitudinal analyses with multiple potential transitions, which can be understood as a concatenation of numerous competing risks models. Even if we have a very large patient population to start with, in routine clinical practice with a wide range of therapeutic options and clinical courses we have pathways that quickly become very branched, heterogeneous and small in frequency. Therefore, such similarity tests, even if they do not initially appear relevant for large amounts of data, can actually get very relevant for large amounts of data, especially for questions relating to pathway similarity.

We conclude mentioning further interesting directions for future research. One is the use of nonparametric methods for the estimation transition intensities [31, 32]. However, a nonparametric approach for testing the hypotheses (10) versus (11) requires the asymptotic distribution of statistics of the form $∥{\hat{\alpha }}_{0j}^{\left(1\right)}-{\hat{\alpha }}_{0j}^{\left(2\right)}{∥}_{\infty }-∥\alpha_{0j}^{\left(1\right)}-\alpha_{0j}^{\left(2\right)}{∥}_{\infty }$ (here ${\hat{\alpha }}_{0j}^{\left(1\right)}$ and ${\hat{\alpha }}_{0j}^{\left(2\right)}$ denote the nonparametric estimates), which is not known up to now. For a first step in this direction, indicating the mathematical difficulties of such an approach in the context of nonparametric regression we refer to Bücher, Dette, and Heinrichs [33].

Another challenging question is the extension of our approach to other target parameters such as transition or occupation probabilities. To illustrate the difficulties, consider, for example, the case, where all transition intensities are constant, that is $\alpha_{0j}^{\left(\ell \right)}\left(t\right)=\theta_{0j}^{\left(\ell \right)}$. In this case, we can calculate the transition probabilities from the transition intensities using the matrix exponential of the transition rate matrix and can consider the hypothesis:

$$H_{0}:d_{\infty }^{\mathbb{P}}=\max_{j=1,\ldots ,k}\max_{t\in \left[0,\tau \right]}|{\mathbb{P}}_{0j}^{\left(1\right)}\left(0,t\right)-{\mathbb{P}}_{0j}^{\left(2\right)}\left(0,t\right)|\geq \Delta$$

In the same way (using the matrix exponential of the estimated transition rate matrix), we obtain an estimator ${\hat{d}}_{\infty }^{\mathbb{P}}$ of $d_{\infty }^{\mathbb{P}}$. However, in order to implement the constrained bootstrap approach we would have to generate data under the constraint $d_{\infty }^{\mathbb{P}}=\Delta$ which cannot be directly translated into a constraint regarding the parameters of the transition intensities.

## Conflicts of Interest

The authors declare no conflicts of interest.

## Supporting information


**Data S1** Supplementary Information.

## Data Availability

The prostate cancer dataset used in the application example cannot be shared due to privacy and ethical restrictions. The code used in the simulation study (Section 3) can be found at https://github.com/kathrinmoellenhoff/Similarity‐of‐competing‐risk‐models.

## 1

Proof of Theorem 2.1 Recall the definition of the vector of parameters $\theta^{\left(\ell \right)}\in {\mathbb{R}}^{p}$ in (6) (ℓ=1,2) and define $\theta ={\left({\left(\theta^{\left(1\right)}\right)}^{⊤}{\left(\theta^{\left(2\right)}\right)}^{⊤}\right)}^{⊤}\in {\mathbb{R}}^{2p}$ as the vector of all parameters in the two competing risk models. Furthermore denote by ${\hat{\theta }}_{0j}^{\left(\ell \right)}$, ${\hat{\theta }}^{\left(\ell \right)}$ and $\hat{\theta }={\left({\left({\hat{\theta }}^{\left(1\right)}\right)}^{⊤}{\left({\hat{\theta }}^{\left(2\right)}\right)}^{⊤}\right)}^{⊤}$ the corresponding maximum likelihood estimators defined by maximizing (5) (or equivalently 7) and define by

(A1)
$${\hat{\alpha }}_{0j}^{\left(\ell \right)}\left(t\right)={\hat{\alpha }}_{0j}^{\left(\ell \right)}\left(t,{\hat{\theta }}_{0j}^{\left(\ell \right)}\right)$$

the corresponding estimators of the transition intensity functions (note that for j=1,…,k, ℓ=1,2 (A1) defines a 2k‐dimensional vector of functions defined on the interval 𝒯=[0,τ]). Then, by Theorem 2 in Borgan [27] it follows that $\sqrt{n}\left(\hat{\theta }-\theta \right)$ converges weakly to a multivariate normal distribution with mean vector 0 and a block diagonal covariance matrix. We now interpret the vectors as stochastic processes on the finite set ℳ={1,…,k}×{1,2} and rewrite this weak convergence as

(A2)
$${\left\{\sqrt{n}\left({\hat{\theta }}_{0j}^{\left(\ell \right)}-\theta_{0j}^{\left(\ell \right)}\right)\right\}}_{\left(j,\ell \right)\in ℳ}⇝{\left\{D\left(j,\ell \right)\right\}}_{\left(j,\ell \right)\in ℳ}$$

Therefore, an application of the continuous mapping theorem (see, e.g., van der Vaart [34]) implies the weak convergence of the process

(A3)
$$\begin{array}{c} {\left\{\sqrt{n}\left(\left(\alpha_{0j}^{\left(1\right)}\left(t,{\hat{\theta }}_{0j}^{\left(1\right)}\right)-\alpha_{0j}^{\left(1\right)}\left(t,\theta_{0j}^{\left(1\right)}\right)\right)-\left(\alpha_{0j}^{\left(2\right)}\left(t,{\hat{\theta }}_{0j}^{\left(2\right)}\right)-\alpha_{0j}^{\left(2\right)}\left(t,\theta_{0j}^{\left(2\right)}\right)\right)\right)\right\}}_{\left(j,t\right)\in 𝒳}\\ ⇝{\left\{G\left(j,t\right)\right\}}_{\left(j,t\right)\in 𝒳} \end{array}$$

in $\ell^{\infty }\left(𝒳\right)$, where 𝒳={1,…,k}×𝒯 and ${\left\{G\left(j,t\right)\right\}}_{\left(j,t\right)\in 𝒳}$ is a centered Gaussian process on 𝒳. Note that this is the analog of the equation (A.7) in Dette et al. [19], and it follows by similar arguments as stated in this paper that

(A4)
$$\begin{array}{l} \sqrt{n}\left(∥{\hat{\alpha }}^{\left(1\right)}-{\hat{\alpha }}^{\left(2\right)}{∥}_{\infty ,\infty }-∥\alpha^{\left(1\right)}-{\hat{\alpha }}^{\left(2\right)}{∥}_{\infty ,\infty }\right)\\ \to \max\left\{\max_{\left(j,t\right)\in {ℰ}^{+}}G\left(j,t\right),\max_{\left(j,t\right)\in {ℰ}^{-}}-G\left(j,t\right)\right\} \end{array}$$

where the vectors ${\hat{\alpha }}^{\left(\ell \right)}$ and ${\hat{\alpha }}^{\left(\ell \right)}$ are defined by ${\hat{\alpha }}^{\left(\ell \right)}\left(t\right)=({\hat{\alpha }}_{0j}^{\left(\ell \right)}{\left(t,{\hat{\theta }}_{0j}^{\left(\ell \right)}\right)}_{j\in \left\{1,\ldots ,k\right\}}$ and $\alpha^{\left(\ell \right)}\left(t\right)=(\alpha_{0j}^{\left(\ell \right)}{\left(t,{\hat{\theta }}_{0j}^{\left(\ell \right)}\right)}_{j\in \left\{1,\ldots ,k\right\}},$ respectively, and

$${ℰ}^{\pm }=\left\{\left(j,t\right)\in \left\{1,\ldots ,k\right\}\times 𝒯:{\hat{\alpha }}_{0j}^{\left(1\right)}\left(t\right)-{\hat{\alpha }}_{0j}^{\left(2\right)}\left(t\right)=\pm ∥{\hat{\alpha }}^{\left(1\right)}-{\hat{\alpha }}^{\left(2\right)}{∥}_{\infty ,\infty }\right\}$$

Note that ${ℰ}^{-}\cup {ℰ}^{+}=ℰ$, where ℰ is defined in (20), and that (A4) is the analog of Theorem 3 in Dette et al. [19] Similarly, we obtain the weak convergence of the bootstrap process and the corresponding statistic, that is

(A5)
$$\begin{array}{c} {\left\{\sqrt{n}\left(\left(\alpha_{0j}^{\left(1\right)}\left(t,{\hat{\theta }}_{0j}^{*\left(1\right)}\right)-\alpha_{0j}^{\left(1\right)}\left(t,{\hat{\hat{\theta }}}_{0j}^{\left(1\right)}\right)\right)-\left(\alpha_{0j}^{\left(2\right)}\left(t,{\hat{\theta }}_{0j}^{*\left(2\right)}\right)-\alpha_{0j}^{\left(2\right)}\left(t,{\hat{\hat{\theta }}}_{0j}^{\left(2\right)}\right)\right)\right)\right\}}_{\left(j,t\right)\in 𝒳}\\ ⇝{\left\{G\left(j,t\right)\right\}}_{\left(j,t\right)\in 𝒳} \end{array}$$

and

$$\begin{array}{c} \sqrt{n}\left(∥{\hat{\alpha }}^{*\left(1\right)}-{\hat{\alpha }}^{*\left(2\right)}{∥}_{\infty ,\infty }-∥{\hat{\alpha }}^{\left(1\right)}-{\hat{\alpha }}^{\left(2\right)}{∥}_{\infty ,\infty }\right)\\ \to \max\left\{\max_{\left(j,t\right)\in {ℰ}^{+}}G\left(j,t\right),\max_{\left(j,t\right)\in {ℰ}^{-}}-G\left(j,t\right)\right\} \end{array}$$

conditionally on $X_{1}^{\left(1\right)},\ldots ,X_{n_{1}}^{\left(1\right)},X_{1}^{\left(2\right)},\ldots ,X_{n_{2}}^{\left(2\right)}$, where ${\hat{\alpha }}^{*\left(\ell \right)}$ is the bootstrap version of ${\hat{\alpha }}^{\left(\ell \right)}$ and ${\hat{\hat{\alpha }}}^{\left(\ell \right)}$ is obtained by the constrained estimates ${\hat{\hat{\theta }}}_{0j}^{\left(\ell \right)}$, that is, ${\hat{\hat{\alpha }}}_{0j}^{\left(\ell \right)}\left(t\right)=\alpha_{0j}^{\left(\ell \right)}\left(t,{\hat{\hat{\theta }}}_{0j}^{\left(\ell \right)}\right)$, j=1,…,k, ℓ=1,2, see also Algorithm 1. This is the analog of statement (A.25) in Dette et al. [19]. Now the statements (A.7) and (A.25) and their Theorem 3 are the main ingredients for the proof of Theorem 4 in Dette et al. [19]. In the present context, these statements can be replaced by (A3), (A5), and (A4), respectively, and a careful inspection of the arguments given in Dette et al. [19] proves the claim of Theorem 2.1.□
