# Supplementary material for: Testing Similarity of Parametric Competing Risks Models for Identifying Potentially Similar Pathways in Healthcare
Source: Stat Med. 2024 Oct 12;43(28):5316–30. doi: 10.1002/sim.10243 (PMC11586916; doi:10.1002/sim.10243)
Supplement: Supplementary file 1 — Data S1 Supplementary Information. [file SIM-43-5316-s001.pdf]

Received: Added at production | Revised: Added at production | Accepted: Added at production

DOI: xxx/xxxx

RESEARCH ARTICLE

Supplementary Material for: “Testing similarity of parametric competing risks models for identifying potentially similar pathways in healthcare”

Kathrin Möllenhoff[1] | **Nadine Binder**[2] | **Holger Dette**[3]

[1]Institute of Medical Statistics and Computational Biology (IMSB), University of Cologne, Cologne, Germany

[2]Institute of General Practice/Family Medicine, Medical Center and Faculty of Medicine, University of Freiburg, Freiburg, Germany

[3]Department of Mathematics, Ruhr University Bochum, Bochum, Germany

Nadine Binder and Kathrin Möllenhoff contributed equally to this work.

Correspondence
Kathrin Möllenhoff, Institute of Medical Statistics and Computational Biology, Faculty of Medicine, University of Cologne, Germany.

Email: kathrin.moellenhoff@uni-koeln.de.

Abstract and Summary

This supplementary material provides further simulation results to investigate the robustness of the method proposed in the main paper. Therefore, two additional simulation scenarios reflecting different levels of misspecification are discussed. We conclude that both slight type I error inflation, i.e. a simulated type I error of 10% at a significance level of 5%, and a conservative behaviour, can occour, depending on how strong the misspecification of the models is. In general, it turns out that for a moderate level of misspecification, the simulated values are very close to the ones obtained from correctly specified models, particularly for increasing sample sizes.

KEYWORDS
multi-state models, parametric competing risks models, small data, similarity, routine clinical data, bootstrap

1 | ROBUSTNESS OF THE TEST PROCEDURE

1.1 | Design

We consider two different settings for the distributions of the transition intensities, which are again driven by the application example given in Section 5 of the main paper. In Scenario 1 we generate the event times according to Scenario 3 of the main paper, i.e. a Gompertz distribution for the first two states and a Weibull distribution for the third state, respectively. Therefore, the intensities of the first two states are given by

$$\alpha_{0j}^{(\ell)}(t, \theta_{0j}^{(\ell)}) = \theta_{0j1}^{(\ell)} \cdot \exp(\theta_{0j2}^{(\ell)} \cdot t),\ j = 1, 2,\ \ell = 1, 2, \tag{1}$$

where $\theta_{0j1}^{(\ell)}$ denotes the scale and $\theta_{0j2}^{(\ell)}$ the shape parameter, respectively, and the transition intensity for the third state is given by

$$\alpha_{03}^{(\ell)}(t, \theta_{03}^{(\ell)}) = \frac{\theta_{032}^{(\ell)}}{\theta_{031}^{(\ell)}} \cdot \left(\frac{t}{\theta_{031}^{(\ell)}}\right)^{\theta_{032}^{(\ell)}-1},\ \ell = 1, 2, \tag{2}$$

where $\theta_{031}^{(\ell)}$ denotes the scale and $\theta_{032}^{(\ell)}$ the shape parameter, respectively.

 © 2023 Copyright Holder Name

We choose the parameters given by the corresponding transition intensities of the application example (see Table 1), resulting in

$$d = \max_{j=1}^{3} \|\alpha_{0j}^{(1)} - \alpha_{0j}^{(2)}\|_{\infty} = \max\{0.0003, 0.0028, 0.0004\} = 0.0028$$

for Scenario 1.

TABLE 1 Estimates of the parameters $\theta_{0j}^{(\ell)}$ of event time distributions for the three transition intensities from competing risks model 1 and competing risks model 2 in the application example. For Gompertz and Weibull, the first value corresponds to the scale and the second value to the shape parameter (see (1) and (2)). Numbers in bold are used in the simulation study.

	Model 1			Model 2		
	$\hat{\theta}_{01}^{(1)}$	$\hat{\theta}_{02}^{(1)}$	$\hat{\theta}_{03}^{(1)}$	$\hat{\theta}_{01}^{(2)}$	$\hat{\theta}_{02}^{(2)}$	$\hat{\theta}_{03}^{(2)}$
Exponential	**0.001**	**0.0011**	**0.004**	**0.0008**	**0.0017**	**0.0009**
Gompertz	**0.002, -0.016**	**0.003,-0.036**	0.0002,0.003	**0.002,-0.018**	**0.006, -0.043**	0.0007,-0.003
Weibull	-0.112, 1304.5	-0.38, 3098.3	**0.097, 2894.8**	-0.12, 1729.8	-0.404, 1595.9	**0.108, 1242.1**

In order to investigate the robustness of the approach, we start with a rather moderate level of misspecification. Precisely, we estimate the first two intensities as Gompertz-distributed, but assume the third one , i.e. $\alpha_{03}^{(\ell)}$, $\ell = 1, 2$, to be constant in both groups.

In a second scenario, we increase the amount of misspecification as follows: we assume Gompertz distributed event times for the first and second state and Weibull distributed event times for the third state, respectively, but the data is generated according to an exponential distribution, i.e. transition intensities are constant. The corresponding parameters are again taken from the application example and displayed in Table 1. The resulting test statistic is given by

$$d = \max_{j=1}^{3} \|\alpha_{0j}^{(1)} - \alpha_{0j}^{(2)}\|_{\infty} = \max\{0.0002, 0.0006, 0.0005\} = 0.0006.$$

The data generating distributions and the assumed ones are summarised in Table 2.

TABLE 2 Summary of the assumed distributions of Scenario 1 and Scenario 2. Misspecified distributions are in bold.

		Model 1			Model 2		
		State 1	State 2	State 3	State 1	State 2	State 3
Scenario 1	Data generating distribution	Gompertz	Gompertz	Weibull	Gompertz	Gompertz	Weibull
	Assumed distribution	Gompertz	Gompertz	**Exponential**	Gompertz	Gompertz	**Exponential**
Scenario 2	Data generating distribution	Exponential	Exponential	Exponential	Exponential	Exponential	Exponential
	Assumed distribution	**Gompertz**	**Gompertz**	**Weibull**	**Gompertz**	**Gompertz**	**Weibull**

According to the main paper, we consider several similarity thresholds Δ and a range of different sample sizes, based on the application example, where $n_1 = 213$ and $n_2 = 482$ patients are observed in the first and second group. Thus we simulate a variety of choices of n_1, n_2, ranging from 200 to 500. Also driven by the application example, we assume administrative censoring with a given follow-up period of 90 days. Consequently, we consider two competing risk models, each with $j = 3$ states over the time range $\mathcal{T} = [0, 90]$. If there is no transition to one of the three states, an individual is administratively censored at these 90 days.

The data in all simulations is generated according to the algorithm described in Beyersmann et al.[1] All simulations have been run using R Version 4.3.0. The total number of simulation runs is $N = 500$ for each configuration and due to computational reasons the test is performed using $B = 250$ bootstrap repetitions. The computation time using an Intel Core i7 CPU with 32GB RAM for one particular dataset with $B = 250$ bootstrap repetitions varies between 3min and 11min, depending on the sample size under consideration.

1.2 | Results of Scenario 1

Scenario 1 reflects a situation of a rather low degree of misspecification, as only the third state is not correctly specified (see Table 2). Table 3 displays the simulated Type I errors and the power for different configurations of the sample size. It becomes obvious that, on the margin of the null hypothesis, i.e. when $\Delta = d$, the type I errors are slightly inflated for small sample sizes, reaching values between 0.056 and 0.108. However, for small and moderate sample sizes, these values are very similar to the simulated type I errors for correctly specified models (see Table 3 of the main paper), while for increasing sample sizes the type I error decreases and converges to the nominal level. A similar conclusion can be drawn regarding the power, which is slightly smaller, but still very close to the values obtained from the correctly specified models. Thus, we conclude that the effect of misspecification, that is a mild type I error inflation and a slight loss of power, is not very strong in this scenario.

TABLE 3
Simulated type I error rates and power of the Test proposed in Algorithm 1 under Scenario 1 of misspecification. Numbers in bold correspond to the margin of the null hypothesis.

	Type I error			Power		
(n_1, n_2)	$\Delta = 0.0006$	$\Delta = 0.0015$	$\Delta = 0.0028$	$\Delta = 0.004$	$\Delta = 0.005$	$\Delta = 0.01$
(200, 200)	0.048	0.062	**0.105**	0.194	0.298	0.860
(250, 300)	0.034	0.054	**0.096**	0.197	0.318	0.965
(300, 300)	0.038	0.056	**0.108**	0.203	0.321	0.964
(250, 450)	0.016	0.034	**0.086**	0.189	0.405	0.990
(300, 500)	0.006	0.024	**0.090**	0.191	0.367	0.998
(500, 500)	0.014	0.032	**0.096**	0.224	0.446	0.996
(1000, 1000)	0.002	0.008	**0.056**	0.360	0.704	0.999

1.3 | Results of Scenario 2

Since in Scenario 2 all transition intensities are assumed to be different from the underlying data generation process, the level of misspecification is higher than in Scenario 1. Table 4 displays the simulated Type I errors and the power. It can be seen that, in contrast to Scenario 1, the behaviour of the test is now conservative, as the type I errors are very small, i.e. below 2%. This effect even increases with increasing sample sizes. Consequently, the power of the test is also very low and is far away from the results obtained from correctly specified models (see Figure 1 in the main paper).

TABLE 4
Simulated type I error rates and power of the Test proposed in Algorithm 1 under Scenario 2 of misspecification. Numbers in bold correspond to the margin of the null hypothesis.

	Type I error		Power	
(n_1, n_2)	$\Delta = 0.0006$	$\Delta = 0.001$	$\Delta = 0.0015$	$\Delta = 0.002$
(200, 200)	**0.010**	0.010	0.038	0.108
(300, 300)	**0.018**	0.028	0.086	0.192
(250, 450)	**0.022**	0.026	0.068	0.202
(300, 500)	**0.016**	0.024	0.080	0.209
(500, 500)	**0.004**	0.012	0.084	0.224

1.4 | Summary

Taking the results of both scenarios into account, we conclude that both slight type I error inflation and a conservative behaviour, can occur, depending on how strong the misspecification of the models is. Of note, this is a general drawback of parametric

approaches and underlines the urgency of fitting the model with great care. However, we observe that for a moderate level of misspecification, the simulated values are very close to the ones obtained from correctly specified models, while the test could suffer from a loss of power in case of strong misspecification. If the sample sizes are large, the performance of the test is very good, even in case of misspecification.

All results presented here rely on simulations based on the real data application which comes along with only few events and, consequently, a very high censoring rate (approx. 80%). Numerically, this makes the estimation procedure in general quiet challenging. Thus, the results presented here are very promising. Further investigations are part of ongoing research.

REFERENCES

1. Beyersmann J, Latouche A, Buchholz A, Schumacher M. Simulating competing risks data in survival analysis. *Statistics in Medicine* 2009; 28(6): 956–971.
